# Upregulated PD-1 Expression Is Associated with the Development of Systemic Lupus Erythematosus, but Not the PD-1.1 Allele of the PDCD1 Gene

**DOI:** 10.1155/2014/950903

**Published:** 2014-04-17

**Authors:** Qingqing Jiao, Cuiping Liu, Ziliang Yang, Qiang Ding, Miaomiao Wang, Min Li, Tingting Zhu, Hua Qian, Wei Li, Na Tu, Fumin Fang, Licai Ye, Zuotao Zhao, Qihong Qian

**Affiliations:** ^1^Department of Dermatology, The First Affiliated Hospital of Soochow University, 188 Shizi Road, Suzhou 215006, China; ^2^Clinical Immunology Laboratory, First Affiliated Hospital, Soochow University, 708 Renmin Road, Suzhou 215007, China; ^3^Department of Dermatology, Soochow University Affiliated Children's Hospital, 303 Jingde Road, Suzhou 215003, China; ^4^Department of Dermatology, First Hospital, Peking University, 8 Xishenku Road, Beijing 100034, China

## Abstract

Systemic lupus erythematosus (SLE) is a multisystem autoimmune disease with complicated genetic inheritance. Programmed death 1 (PD-1), a negative T cell regulator to maintain peripheral tolerance, induces negative signals to T cells during interaction with its ligands and is therefore a candidate gene in the development of SLE. In order to examine whether expression levels of PD-1 contribute to the pathogenesis of SLE, 30 patients with SLE and 30 controls were recruited and their PD-1 expression levels in peripheral blood mononuclear cells (PBMCs) were measured via flow cytometry and quantitative real-time-reverse transcription polymerase chain reaction (RT-PCR). Also, whether PD-1 expression levels are associated with the variant of the SNP rs36084323 and the SLE Disease Activity Index (SLEDAI) was studied in this work. The PD-1 expression levels of SLE patients were significantly increased compared with those of the healthy controls. The upregulated PD-1 expression levels in SLE patients were greatly associated with SLEDAI scores. No significant difference was found between PD-1 expression levels and SNP rs36084323. The results suggest that increased expression of PD-1 may correlate with the pathogenesis of SLE, upregulated PD-1 expression may be a biomarker for SLE diagnosis, and PD-1 inhibitor may be useful to SLE treatment.

## 1. Introduction

Systemic lupus erythematosus (SLE) is a progressive autoimmune disease with a wide range of immunological abnormalities [1]. It is characterized by an immune response against nucleus components, but the etiopathology is not clearly understood yet. Multiple genetic factors relating to SLE have been identified [2–4], which suggest that both immune disorder and genetic factors may play important roles during the SLE process.

The protein programmed death 1 (PD-1), a negative costimulatory molecule, belongs to the CD28 superfamily and is expressed on the surface of activated human CD4^+^ and CD8^+^ T cells, B cells, natural killer (NK) cells, activated monocytes, myeloid cells, and CD4^−^CD8^−^T cells from the thymus [5, 6]. As an immune inhibitory receptor, PD-1 interacts with its ligands, PD-L1 and PD-L2, which can suppress lymphocyte activation and cytokine production [7]. Current concepts regarding PD-1/PD-L pathway are categorized into immune dysfunction associated with SLE in humans [8]. In addition, it was reported that PD-1 gene polymorphisms were involved in the development of autoimmune diseases, such as SLE, rheumatoid arthritis, and Graves' disease [9]. However, until now, only few studies have reported a possible link between PD-1 gene polymorphisms and SLE [8, 10–12]. Due to the existence of racial and regional differences in SNPs in PD-1, it is very important to study the relevance of PD-1 to SLE susceptibility in the Chinese Han population, which could also bring more evidence to the connections among alleles and disease in SLE.

Among single nucleotide polymorphisms (SNPs) in the human PD-1 gene (PDCD1) region, PD-1.1 G/A (rs36084323) was reported to have connection with autoimmune diseases [13]. Previous studies suggested that nonfunctional SNPs could affect gene function through haplotype tagging [14]. In this work, PD-1.1 G/A (rs36084323), a nonfunctional SNPs in PDCD1, was studied in a Chinese Han population, aiming to explore whether PD-1 expression was related to the variant of the SNP PD-1.1 G/A (rs36084323) and SLE Disease Activity Index (SLEDAI).

## 2. Materials and Methods

### 2.1. Patients and Controls

This study was performed on 30 cases of Chinese Han population fulfilling the revised criteria for SLE from American College of Rheumatology 1997 [15, 16] (27 women; 3 men; mean ± SD age, 43.432 ± 14.675) and compared with 30 age-matched controls (27 women; 3 men; mean ± SD age, 41.520 ± 10.478). SLEDAI [17] was taken for each patient at the time of recruitment. The protocol of this study was approved by the ethics committee of the institution involved, and informed consents for genetic studies were obtained from all subjects.

### 2.2. Flow Cytometry Analysis

Flow cytometry was performed using 50 *μ*L of EDTA-treated peripheral blood incubated for 30 min at 4°C with fluorochrome-labeled monoclonal antibodies (mAbs): anti-CD4-FITC (Beckman), anti-CD8-FITC (Beckman), anti-CD56-FITC (Beckman), and anti-PD-1-PE (BioLegend). Erythrocyte lysis and cell fixation were carried out using OptiLyse C Lysing Solution (Beckman). Treated blood samples passed through the Coulter Epics XL Flow cytometer (Beckman), and the relevant data were acquired and accordingly examined. Data analysis was accomplished by FlowJo software (Tree Star, Ashland).

### 2.3. Real-Time RT-PCR Analysis

PBMCs were separated from fresh blood of patient and control groups in order to analyze the mRNA of the PD-1. Total cellular RNA was isolated by Trizol (Invitrogen, USA). After quantification, 1 *μ*g of total cellular RNA was used to conduct reverse transcription with Promega RT kit (A3800) and an oligo (dT) primer. PCR was completed in a 50 *μ*g reaction system containing 200 nM PD-1 primers (Table 1), 120 nM TaqMan probe, and premix Ex Taq (Takara, Dalian, China). Samples were amplified in the Applied Biosystems 7900 HT Fast real-time PCR System (CA, USA) for 40 cycles under the following conditions: denaturation for 10 s at 95°C, anneal and extension for 40 s at 60°C. The expression level of the GAPDH was evaluated as an internal control.

### 2.4. Genotyping

Blood DNA was extracted using Flexi Gene DNA kits (QIAGEN, Germany) according to the manufacturer's instructions, and the DNA samples were then stored at −20°C. DNA fragments spanning PD-1.1 G/A (rs36084323) were amplified by polymerase chain reaction (PCR), and the products were gel-purified and sequenced. Then the sequencing data were applied for Genotyping of SNP (rs36084323).

### 2.5. Statistics

The data of PD-1 mRNA expression levels in PBMCs from all subjects were compared by the Mann-Whitney *U*-test. The genotype frequency of SNP rs36084323 was tested for Hardy-Weinberg equilibrium separately for SLE patients group, while the *P* values > 0.05 for all subjects in control group. Genotypes were compared using the Mann-Whitney *U*-test, and the relation between PD-1 mRNA expression level and the SLEDAI score was examined by Spearman's correlation coefficient rank test. All analyses were processed using SPSS16.0. Data were presented as mean ± SD. *P* value < 0.05 (two-tailed) was considered as statistically significant.

## 3. Results

### 3.1. PD-1 Level Is Upregulated in PBMCs from SLE Patients Compared with Controls

**Figure 1:**
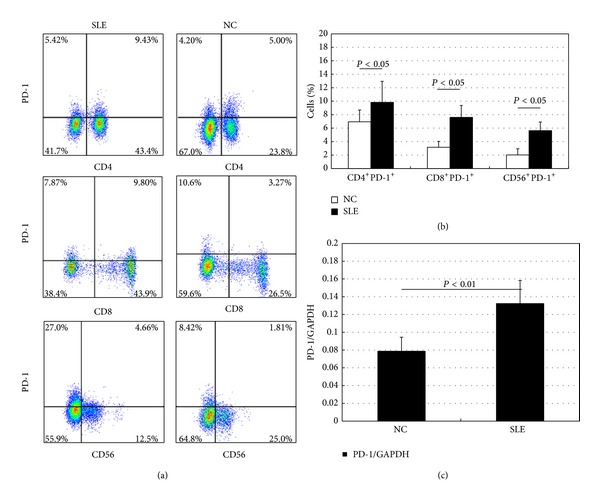
Increased basal programmed death 1 (PD-1) expression in PBMCs from SLE patients. (a) Representative flow cytometry analysis of PD-1 expression on CD4^+^, CD8^+^, and CD56^+^ T cells in SLE patients and normal healthy controls (NC); (b) upregulated expression of PD-1 on CD4^+^, CD8^+^, and CD56^+^ T cells from SLE patients, as compared with those from NC; (c) mRNA expression of PD-1 in PBMCs from SLE patients. Horizontal bars indicate the mean ± SD.

Initially PD-1 protein and mRNA expression levels in all PBMCs samples were examined by flow cytometry and real-time RT-PCR. Flow cytometry analysis results demonstrated that the mean fluorescence intensity (MFI) of PD-1 was higher on CD4^+^ T cells, CD8^+^ T cells, and CD56^+^ T cells from PB samples of SLE patients compared to those of controls (Figures 1(a) and 1(b)). In addition, it is shown that the mean PD-1 mRNA expression levels increased in SLE patients' samples compared with controls' (Figure 1(c)).

### 3.2. SLEDAI Is Significantly Related to the Upregulated PD-1 Expression

**Figure 2:**
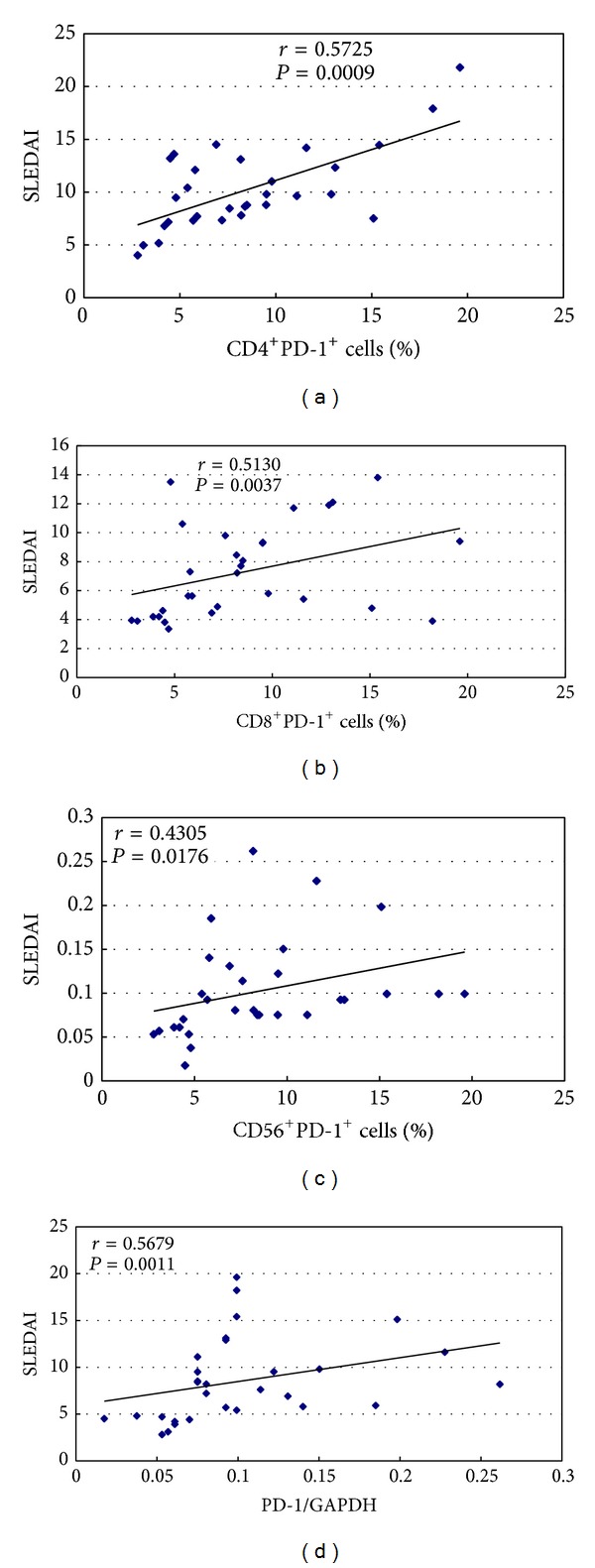
Correlation of upregulated PD-1 expression levels with SLEDAI in PBMCs. The association of SLEDAI with upregulated PD-1 expression on CD4^+^ T cell (a), CD8^+^ T cell (b), CD56^+^ T cell (c), and mRNA expression of PD-1 in PBMCs (d).

In order to determine whether upregulated PD-1 expression is related to SLEDAI, correlation analysis was carried out. The results have shown that SLEDAI scores were significantly related to upregulated PD-1 expression on CD4^+^ T cells, CD8^+^ T cells, CD56^+^ T cells, and increased PD-1 mRNA expression levels in PBMCs from PB samples of SLE patients (Figure 2), which indicates that upregulated PD-1 expression may be involved in the pathogenesis of SLE.

### 3.3. The Variant of the SNP rs36084323 Is Not Related to Upregulated PD-1 Expression

**Figure 3:**
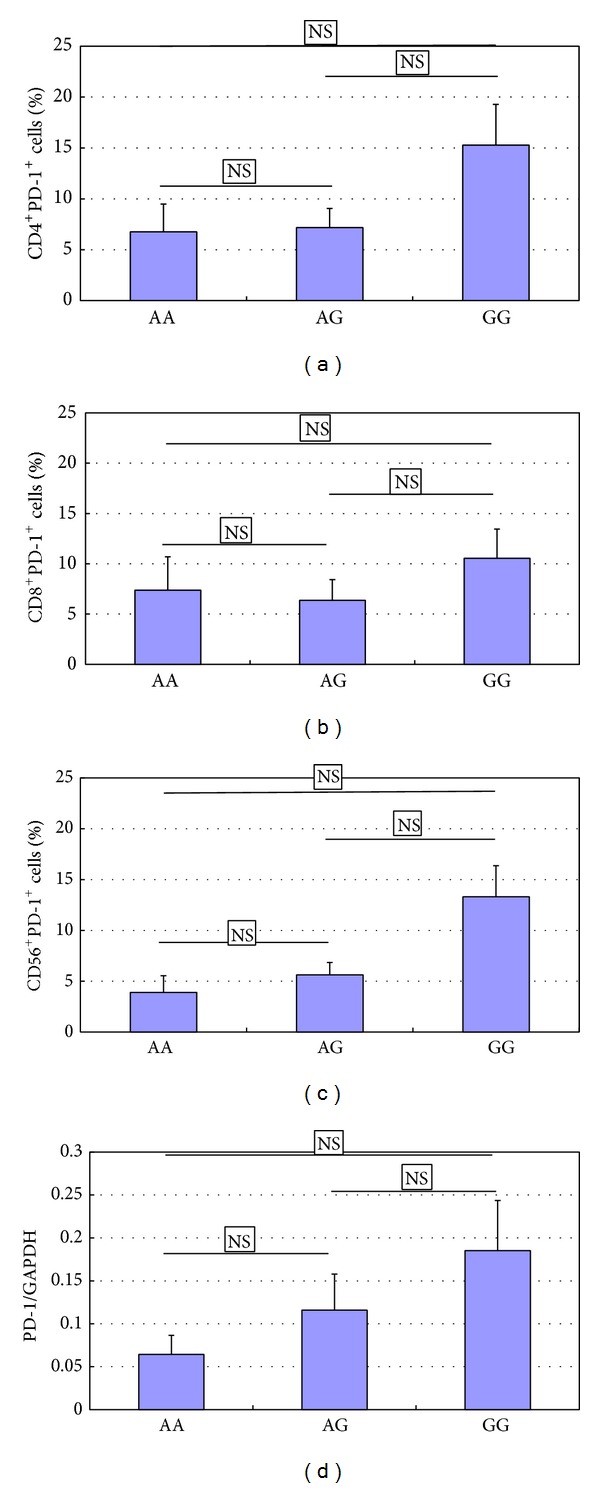
Analysis of PD-1 expression levels in SLE patients with different genotypes of rs36084323. Increased levels of PD-1 levels in PBMC of SLE (*n* = 30) patients, as compared with those from NC (*n* = 30). The patients with GG genotype (*n* = 11) exhibit higher PD-1 expression levels than those with AG (*n* = 15) and AA genotype (*n* = 4). Horizontal bars indicate the mean ± SD.

To explore whether the variant of SNP rs36084323 was related to the upregulated PD-1 expression or not, the variant of SNP rs36084323 was genotyped. Results indicate that there was no connection between the variant of SNP rs36084323 and upregulated PD-1 expression (Figure 3).

## 4. Discussion

In this study, we demonstrated that PD-1 expression levels in PBMCs from SLE patients were significantly higher than those in control group. Also, significant relationship was found between SLEDAI scores and upregulated PD-1 expression in PBMCs from PB samples of SLE patients. However, no obvious difference was revealed between PD-1 expression levels and SNP rs36084323. Results show that increased level of PD-1 expression in PBMCs rather than SNP rs36084323 is associated with the development of SLE, and this discovery is presented for the first time. These findings provide more evidence to support the theory that upregulated PD-1 expression may be involved in the pathogenesis of SLE.

SLE is a chronic inflammatory disease of generalized autoimmunity and is characterized by B cell hyperactivity and abnormally activated T cells [1]. PD-1 can be expressed on activated T cells, B cells, and myeloid cells and is considered to play an important role in the regulation of peripheral tolerance [18]. Mice deficient for PD-1 have developed a lupus-like syndrome, with arthritis and glomerulonephritis as phenotypes [19]. In this study, increased expression of PD-1 in PBMCs is found to have significant relationship with SLEDAI scores, and the results suggest that PD-1 is involved in the development of SLE. Although the detailed etiology is still unclear, many genes are considered to have connections with the pathogenesis of SLE. At present, the programmed cell death 1 gene (PDCD1, also called PD-1) was one of the top candidates linking to the disease [20]. Thus, it is therefore necessary to study the interconnection between polymorphisms in PDCD1 and SLE.

135 SNPs (found in the National Center for Biotechnology Information (NCBI) Entrez SNP database) have been identified in the human PDCD1 region. Among them, PD-1.1, PD-1.3, PD-1.5, and others are considered to have connection to autoimmune diseases [21]. PD-1.1 polymorphism is located in the promoter region (position −538 from transcription start site). Previous studies have shown that PD-1.1 G/A (rs36084323) is common in the Chinese Han population (49%), but it is very rare in Europeans (1%) [20], which may indicate that Chinese Han population is more susceptible to SLE. In this study, no connection was found between SNP PD-1.1 G/A (rs36084323) and increased expression of PD-1 expression in PBMCs from SLE patients (*P* > 0.05), and the reason might be the limited sample size used in this study or some yet unidentified reason. However, it is observed that frequencies of the GG and AG genotype allele in SNP PD-1.1 were higher in SLE patients when compared with AA in our patients' population. In addition, PD-1.1 is located within the promoter region of PD-1. This SNP has no function, and further study is required to explore its exact role in the development of SLE.

## 5. Conclusions

In conclusion, increased expression of PD-1 in PBMCs from SLE patients was significantly related to SLEDAI scores rather than SNP rs36084323. The presented results provide more evidence to support that upregulated expression of PD-1 might be correlated with the pathogenesis of SLE.

## Figures and Tables

**Table 1:** Primers of SNP PD-1.1 used for sequencing.

Maker name	SNP	rs number	Location	PCR primers
PD-1.1	−538 G/A	rs36084323	promoter	Forward: 5′-CCCGTCAGGCTGTTGCA-3′
				Reverse: 5′-CCCTCTTCCTCCACATCCAC-3′

SNP: single nucleotide polymorphism; PCR: polymerase chain reaction.

## References

[1] Paz Z, Tsokos GC (2013). New therapeutics in systemic lupus erythematosus. *Current Opinion in Rheumatology*.

[2] Costa-Reis P, Sullivan KE (2013). Genetics and epigenetics of systemic lupus erythematosus. *Current Rheumatology Reports*.

[3] da Silva Fonseca AM, de Azevedo Silva J, Pancotto JA (2013). Polymorphisms in STK17A gene are associated with systemic lupus erythematosus and its clinical manifestations. *Gene*.

[4] Cui Y, Sheng Y, Zhang X (2013). Genetic susceptibility to SLE: recent progress from GWAS. *Journal of Autoimmunity*.

[5] Araki K, Youngblood B, Ahmed R Programmed cell death 1-directed immunotherapy for enhancing T-cell function.

[6] Kasagi S, Kawano S, Kumagai S (2011). PD-1 and autoimmunity. *Critical Reviews in Immunology*.

[7] Fife BT, Pauken KE (2011). The role of the PD-1 pathway in autoimmunity and peripheral tolerance. *Annals of the New York Academy of Sciences*.

[8] Okazaki T, Wang J (2005). PD-1/PD-L pathway and autoimmunity. *Autoimmunity*.

[9] Gianchecchi E, Delfino DV, Fierabracci A (2013). Recent insights into the role of the PD-1/PD-L1 pathway in immunological tolerance and autoimmunity. *Autoimmunity Reviews*.

[10] Kristjansdottir H, Steinsson K, Gunnarsson I, Gröndal G, Erlendsson K, Alarcón-Riquelme ME (2010). Lower expression levels of the programmed death 1 receptor on CD4+CD25+ T cells and correlation with the PD-1.3A genotype in patients with systemic lupus erythematosus. *Arthritis and Rheumatism*.

[11] Bertsias GK, Nakou M, Choulaki C (2009). Genetic, immunologic, and immunohistochemical analysis of the programmed death 1/programmed death ligand 1 pathway in human systemic lupus erythematosus. *Arthritis and Rheumatism*.

[12] Wang SC, Chen YJ, Ou TT (2006). Programmed death-1 gene polymorphisms in patients with systemic lupus erythematosus in Taiwan. *Journal of Clinical Immunology*.

[13] Greenwald RJ, Freeman GJ, Sharpe AH (2005). The B7 family revisited. *Annual Review of Immunology*.

[14] Ming ZJ, Hui H, Miao M, Qiu YH, Zhang XG (2013). Polymorphisms in PDCD1 gene are not associated with aplastic anemia in Chinese Han population. *Rheumatology International*.

[15] Tan EM, Cohen AS, Fries JF (1982). The 1982 revised criteria for the classification of systemic lupus erythrematosus. *Arthritis and Rheumatism*.

[16] Hochberg MC (1997). Updating the American College of Rheumatology revised criteria for the classification of systemic lupus erythematosus. *Arthritis and rheumatism*.

[17] Bombardier C, Gladman DD, Urowitz MB, Caron D, Chang CHC (1992). Derivation of the SLEDAI: a disease activity index for lupus patients. *Arthritis and Rheumatism*.

[18] Francisco LM, Sage PT, Sharpe AH (2010). The PD-1 pathway in tolerance and autoimmunity. *Immunological Reviews*.

[19] Nishimura H, Nose M, Hiai H, Minato N, Honjo T (1999). Development of lupus-like autoimmune diseases by disruption of the PD-1 gene encoding an ITIM motif-carrying immunoreceptor. *Immunity*.

[20] Prokunina L, Castillejo-López C, Öberg F (2002). A regulatory polymorphism in PDCD1 is associated with susceptibility to systemic lupus erythematosus in humans. *Nature Genetics*.

[21] Prokunina L, Padyukov L, Bennet A (2004). Association of the PD-1.3A allele of the PDCD1 gene in patients with rheumatoid arthritis negative for rheumatoid factor and the shared epitope. *Arthritis and Rheumatism*.

